# Seed Heteromorphism: An Important Adaptation of Halophytes for Habitat Heterogeneity

**DOI:** 10.3389/fpls.2018.01515

**Published:** 2018-10-17

**Authors:** Ranran Liu, Lei Wang, Mohsin Tanveer, Jie Song

**Affiliations:** ^1^Shandong Provincial Key Laboratory of Plant Stress, College of Life Science, Shandong Normal University, Jinan, China; ^2^State Key Laboratory of Desert and Oasis Ecology, Xinjiang Institute of Ecology and Geography, Chinese Academy of Sciences, Ürümqi, China; ^3^School of Land and Food, University of Tasmania, Hobart, TAS, Australia

**Keywords:** dormancy, halophyte, heteromorphism, salinity, seed germination

## Abstract

Seed germination is a very critical and important step for seedling establishment under saline environments, as high level of salinity in the soil can prevent seed germination. However halophytes exhibit an interesting mechanism to cope with salt stress. Many halophytes produce heteromorphic seeds, which have different dormancy and germination behavior under saline conditions. This characteristic is related to the structural and physiological differences among heteromorphic seeds. It was unclear that how heteromorphic seeds differently accumulate organic and inorganic substances under saline conditions, and what are the physiological and molecular mechanisms involved in the production of heteromorphic seeds, and in the development of transgenerational plasticity in heteromorphic seeds. In the current brief review, dormancy and germination and the possible role of seed coat and storage compounds in this process of heteromorphic seeds development have been discussed. Moreover, the role of maternal effects on heteromorphic seeds production under saline environments and growth and reproduction capability of the descendants from them have been highlighted.

## Introduction

**FIGURE 1:**
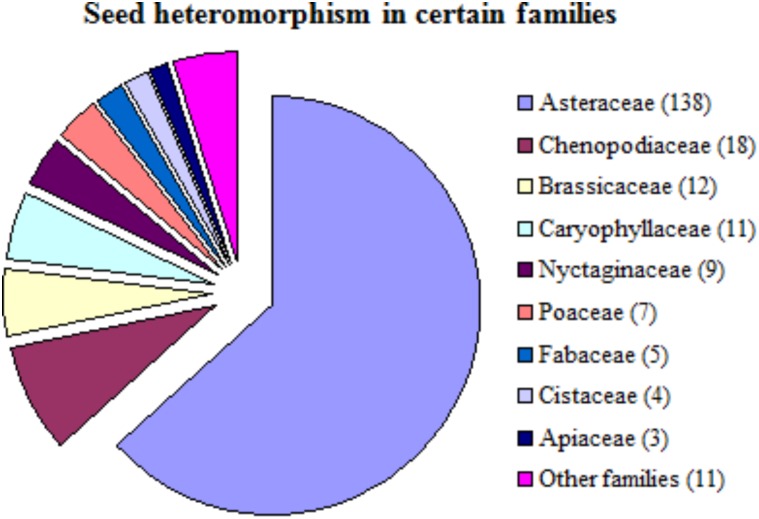
Nature of seed heteromorphism in different plant families. Numbers in parenthesis after the family are showing number of species, which showed seed heteromorphic response (adapted from Imbert, 2002).

More than 950 million hectares of land worldwide are salt-affected, which account for about 10% of total land worldwide (Shabala, 2013). Salinity affects utilization of soil and ground water, particularly in arid and semiarid regions (Rozema and Flowers, 2008). Moreover, inevitable conversion of arable land area into urban land has further forced to start the agricultural production in marginal areas (Shabala, 2013). One strategy to deal with salt-affected land is to utilize halophytes for reclamation and productivity (Rozema and Flowers, 2008; Song and Wang, 2015). Halophytes are plants which can survive and reproduce in saline environments where the salt concentration is higher than 200 mM NaCl (Flowers and Colmer, 2008). Some halophytes are being used as edible plants such as *Chenopodium quinoa* and some can also be used for removing extra salts from salt affected lands such as *Suaeda salsa* (Song and Wang, 2015). Halophytes can survive and propagate in a very complex way to ensure the production of their subsequent generation. Halophytes can adapt to salinity with numerous adaptive mechanisms such as ion exclusion by the roots (Munns and Tester, 2008; Song et al., 2011; Flower and Colmer, 2015; Chen et al., 2016; Liu et al., 2018), maintaining ion homeostasis in the leaves (Munns and Tester, 2008; Yang et al., 2010; Shao et al., 2014; Tanveer et al., 2018), secretion of toxic ions such as Na^+^ and Cl^−^ through the salt glands (Ding et al., 2010; Deng et al., 2015; Feng et al., 2015; Yuan et al., 2015, 2016a,b; Leng et al., 2018; Tanveer and Shabala, 2018). However, halophytes can not adopt these mechanisms during seed germination. Seed heteromorphism is generally considered as an adaptive strategy that helps plants to survive in changeable and unpredictable environments, especially in deserts and/or in high saline soils (Gul et al., 2013; Xu et al., 2016). Seed germination is critical in plant life cycle, especially for halophytes in saline environments, as high levels of salinity in the soil can prevent/limit seed germination (Guo et al., 2012a,b; Gul et al., 2013; Zhang et al., 2015). Moreover, heteromorphic seed production under salt stress could be a kind of bet-hedging strategy for their next generations, becoming naturally acclimated to adverse environment (Imbert, 2002; Tanveer and Shah, 2017). Many halophytic species show seed dimorphism or polymorphism (**Figure 1** and **Table 1**) and among these heteromorphic species, most of halophytic species are annual, only a few species belong to perennials such as *Arthrocnemum indicum* (Khan and Gul, 1998), and *Tamarix ramosissima* (Yan et al., 2011). Up to now, many published papers have been focused in characterizing the germination pattern and dormancy mechanism in heteromorphic seeds of halophytes, especially in Amaranthaceae. Recently, it has been shown in a non-halophytic plant species- *Aethionema arabicum* that seed dimorphism was associated with several physiological and anatomical features and gene expression levels between the fruit and seed morphs (Lenser et al., 2016) and latter it was found that cytokinin, and transcripts of *BRANCHED1* gene were involved in governing fruit dimorphism (Lenser et al., 2018). In this brief review, germination pattern of different dimorphs and factors which influence seed germination of those dimorphs have been discussed. Moreover, role of seed dormancy as adaptive strategy in halophytes under saline conditions has also been discussed.

**Table 1:** Reported examples of heteromorphic seed production and their different heteromorphs in halophytes under saline conditions.

Species	Morphs	Habitat	Reference
*Atriplex centralasiatica*	Brown; Black	Desert, saline soil	Li et al., 2008
*Atriplex triangularris*	Large; Medium; Small	Inland and coastal marshes	Khan and Ungar, 1984
*Atriplex aucher*	Brown; Black	Desert, saline soil	Wei et al., 2003
*Atriplex patens*	Brown; Black	Saline soil	He and Li, 1995
*Atriplex micrantha*	Brown; Black	Desert, saline soil	He and Li, 1995
*Atriplex rosea*	Brown (larger); Black (smaller)	Salt marshes	Khan et al., 2004
*Atriplex sagittata*	Large; Medium; Small	Salt steppe and riparian habitats	Mandák and Pyšek, 2005
*Atriplex prostrata*	Large; Small	Inland and coastal marshes	Carter and Ungar, 2003
*Atriplex inflata*	Brown; Black	Saline soil	Abdallah et al., 2011
*Suaeda glauca*	Brown; Black	Saline soil	Duan et al., 2018
*Suaeda linifolia Suaeda paradoxa Suaeda kossinskyi*	Brown; Black	Saline soil	Zhang, 2010
*Suaeda splendens*	Brown; Black	Saline soil	Redondo-Gómez et al., 2008
*Suaeda salsa*	Brown (larger); Black (smaller)	Saline soil	Li et al., 2005
*Suaeda aralocaspica*	Brown (larger), Black (smaller)	Salinized desert	Wang et al., 2008
*Suaeda moquinii*	Brown (soft); Black (hard)	Salt marshes	Khan et al., 2001
*Suaeda acuminata*	Brown; Black	Salinized desert	Wang L. et al., 2012
*Salicornia europaea*	Large; Small	Inland and coastal marshes	Ungar, 1979
*Salicornia ramosissima*	Large (central); Small (lateral)	Saline soil, coastal marshes	Ameixa et al., 2016
*Chenopodium album*	Brown (larger); Black (smaller)	Light-saline soil	Yao et al., 2010
*Halogeton glomeratus*	Green; Yellow	Inland salt deserts	Yu, 2008
*Tamarix ramosissima*	Spring; Summer	Piedmont Gobi desert, valleys, saline soil	Yan et al., 2011
*Salsola komarovii*	Long winged; Short winged	Coastal regions	Yamaguchi et al., 1990
*Salsola affinis*	^∗^Types A, B, C	Gravel desert, saline soil	Wei et al., 2007
*Salsola ferganica*	Large with or without winged perianth (WP); Medium with or without WP; Small with or without WP	Cold desert	Ma et al., 2018
*Arthrocnemum indicum*	Brown; Black	Inland and coastal marshes	Khan and Gul, 1998
*Cakile edentula*	Large; Small	Coastal dune	Zhang, 1993

*^∗^Type A fruits have lignified perianths with long wings and green utricles; Type B fruits have lignified perianths with short wings, or no wings, and green utricles; Type C fruits have tepals without wings and yellow utricles.*

## Germination of Heteromorphic Seeds Under Salt Stress

### Role of Seed Size and Seed Parental Material

Development of relatively large embryo or cotyledon is an important feature of certain halophytes to ensure optimum seedling establishment under high saline conditions (Zhang et al., 2010; Li et al., 2012; Zhou et al., 2014). Studies showed that halophytic species with bigger seed size exhibited better germination percentage under saline conditions (Song et al., 2008, 2016; Yao et al., 2010; Zhang et al., 2010; Gul et al., 2013; Wang et al., 2018). Published literature reported that halophytes produce larger seed with big storage capacity under salt stress. For example, certain halophytic plants such as *S. salsa* produce large seed and transfer more photo-assimilates to those larger seed in order to ensure optimum germination for next generation (Guo et al., 2015; Wang et al., 2015; Zhao et al., 2018a). Nonetheless, molecular identification of such mechanism has not been reported therefore, more research is required to further elaborate this mechanism.

Seed maternal environment may also influence numerous traits relating to seed germination (Wang L. et al., 2012; Yang et al., 2015). Stress conditions may provide signals to mother plants to produce subsequent generation with different phenotypes (due to trans-generational effects) as an adaptive strategy to survive under stress conditions. An experiment was performed to examine such maternal effects and dimorphic seed production in *Suaeda corniculata* subsp. *mongolica* under different environmental conditions (Yang et al., 2015). They found that seed maternal effects play an important role in the ecological diversity and in salt stress tolerance seed production under salt stress conditions along with numerous temporal and spatial variations (Yang et al., 2015). High concentrations of salts may also provide signals to plants to produce dimorphic seeds with better seed germination. For instance, according to Wang et al. (2015), with increasing salt stress levels, *S. salsa* produced more brown seeds with better salt stress tolerance capacity as compared with black seeds. Phosphatidylglycerol (PG) is a glycerophospholipid. PG improves salt stress tolerance in seeds via increasing high production of unsaturated fatty acids, for instance, *Arabidopsis*, overexpressed with *SsGPAT* gene, exhibited high contents of unsaturated fatty acid and showed significant salt stress tolerance (Sui et al., 2017). It was found that PG contents were higher in young seedlings derived from brown seeds from high-salinity maternal plants (Zhou et al., 2016), and an increased PG level may be related to salt tolerance in *S. salsa* (Sui et al., 2010; Cheng et al., 2014), and in *Thellungiella halophila* (Sui and Han, 2014). The hereditary evidences relating to varied salt tolerance capability of dimorphic seeds produced from salinity stressed maternal plants, need to be further tested.

### Role of Organic Osmolytes in Seed Germination

Organic osmolytes play very important role in stress alleviation (Anjum et al., 2016, 2017a). Halophytes (for example *S. salsa*) produce/accumulate more organic osmolytes such as starch, soluble sugar, and protein in their large seeds as compared with small seeds to ensure optimum seed germination under salt stress (Guo et al., 2015, 2018; Wang et al., 2015; Zhao et al., 2018a). These osmolytes such as soluble sugars can help in ROS detoxification or can also act as signaling compounds that may trigger other stress alleviation mechanisms (Gibson, 2005; Couée et al., 2006; Anjum et al., 2017b).

Among different kinds of osmolytes, betaine is an important osmotic regulation substance, and accumulation of betain could be a defensive strategy by halophytes to cope with high salt stress levels. Halophytes can produce salt tolerant seeds with more betain and can ensure good seed germination under salt stress (Tanveer and Shah, 2017). Expression analysis of betaine aldehyde dehydrogenase gene (*SsBADH*) in *S. salsa* showed that brown seeds have higher expression of *SsBADH* and exhibit better seed germination as compared with black seeds (Xu et al., 2017). Proline is generally regarded as another important osmoregulator (Flowers and Colmer, 2008), nonetheless studies showed that proline also acts as signaling compound, which induces delayed germination process by keeping embryo in resting state under stress conditions (Thakur and Sharma, 2005). According to Xu et al. (2017) proline not only increases osmotic adjustment in brown seeds but also protects seeds from being damaged under salt stress.

Soluble sugars play very crucial role in the process of osmotic adjustment during seed germination under salt stress. It was reported that halophytes (e.g., *S. salsa*) accumulate more soluble sugars in brown seeds as compared with the black seeds (Guo et al., 2015; Zhao et al., 2018a). The embryos of large seeds, especially the cotyledons, are better developed than that of small seeds, such as *S. salsa* (Song et al., 2008). The embryos of fresh premature seeds contained chlorophyll and the embryos could take the function of photosynthesis (Li et al., 2012). Therefore, better developed embryos in brown seeds may help brown seeds to provide more soluble sugar during seed germination, and soluble sugar may play an important role in their rapid germination for brown seeds compared to black seed in *S. salsa*.

Phytohormones sometimes also act as osmoregulaters, as in an endogenous hormones analysis, it was examined that brown seeds exhibited more ABA (abscisic acid), IAA (indole-3-acetic acid), and ZR (zeatin riboside) as compared with black seeds, and these hormones acted as osmoregulater (Wang et al., 2015). According to Karssen et al. (1983), during seed development ABA induces the accumulation of numerous storage proteins, which contribute in increasing the embryo size and seed weight of brown seeds under salt stress (Wang et al., 2015).

### Role of Inorganic Ions in Seed Germination

Besides organic compounds, inorganic ions also improve seed germination by improving imbibition process during seed germination (Song et al., 2005; Xu et al., 2017). In the brown seeds of *S. salsa*, the contents of Na^+^, K^+^, Cl^−^, and Ca^2+^ were higher as compared with black seeds, and may be due to the higher activity of relative transporters in brown seeds such as vacuolar Na^+^/H^+^ antiporter (NHX), potassium transporter (HAK), chloride channel protein (CLC) and Ca^2+^/H^+^ antiporter at tonoplast (CAX) as compared with black seeds which were involved in maintaining ion homeostasis and improving water uptake for seeds during germination under salt stress (Xu et al., 2017). It is well-documented that maintaining ion homeostasis plays a critical role in plant salt tolerance (Munns and Tester, 2008; Chen et al., 2010; Zhao et al., 2010; Han et al., 2011, 2012; Flower and Colmer, 2015). It is not surprising that brown seeds had higher germination rate than black seeds under high saline conditions (Song et al., 2008).

The micronutrients such as iron, zinc, or copper are essential micronutrients for all living organisms (Waters and Sankaran, 2011). Halophytes accumulate more micronutrients in salt tolerant seeds than salt sensitive seeds. For instance, *S. salsa* accumulates high amount of Fe, Mn and Zn, especially Fe in brown seeds as compared with black seeds, i.e., the Fe contents were 6.6 and 2.2 μmol g^−1^ DW (rather than mmol g^−1^ DW) in brown and black seeds, respectively (Zhao et al., 2018b). Micronutrients pass through leaves and seed covering tissues to reach seeds. Therefore, certain genes with increased expression in these tissues during seed fill may be important for translocation of relative micronutrients to seeds. However no molecular evidence has been reported so far, showing how seed-heteromorphic halophytes differently load or unload or transfer micronutrients in heteromorphic seeds in the same maternal environment.

### Role of Seed Coat in Adaptation of Heteromorphic Seeds to Saline Environments

**FIGURE 2:**
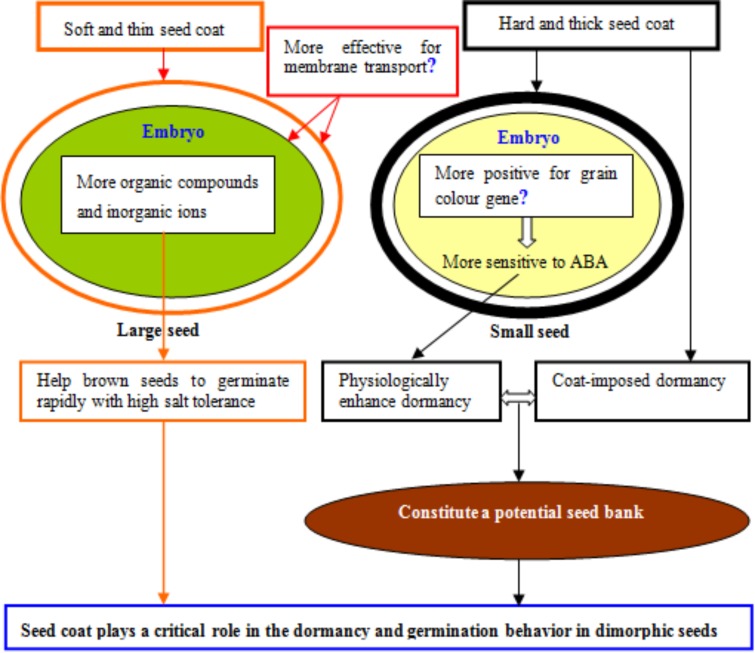
Possible role of seed coat in the dormancy and germination behavior of heteromorphic seeds in halophytes, using dimorphic seeds.

Seed coat is very crucial during seed germination, and seed survival (**Figure 2**; Souza and Marcos-Filho, 2001). Few studies reported the structure and chemical reactions in the seeds of halophytic species may be related to their dormancy/germination behavior (Souza and Marcos-Filho, 2001; Himi et al., 2002; Shepherd et al., 2005; Yang et al., 2012). Normally, dormant seeds have hard seed coat, and due to hard seed coat, dormancy persists until a portion of the coat becomes ruptured and permeable to water, and then seeds start imbibition process. Seeds may lose this coat-imposed dormancy during a several-year period in the field (Egley et al., 1986). In seed coat, accumulation of numerous hydrophobic compounds such as lipids, callose, and lignin in the palisade cells, makes seed impermeable to water and limit seed germination (Kelly et al., 1992). Moreover seed coat permeability in dimorphic seeds is linked with the seed coat thickness, which is usually lower in non-dormant (tolerant) seeds as compared with dormant (sensitive) seeds. Seed coat permeability also depends on the accumulation of phenolics contents, suberin and cellulose in palaside cells (Serrato-Valenti et al., 1990). However it is still unknown that how these substances interact with water permeability, therefore physiological and molecular evidences in the process of seed coat development in heteromorphic seeds are needed to explore in future work. Delay in germination could be an adaptive strategy to stay under shade until external environment becomes favorable for seed germination. Li et al. (2005) showed that dormant seeds such as black seeds in *S. salsa* take up water more slowly than non-dormant brown seeds (Li et al., 2005). Recently, it has been showed that mucilage in seed coat also increases dormancy and delays germination by reducing the diffusion of oxygen through seed coat (Yang et al., 2012).

In *S. salsa*, it has been noted that hard seed coat in black seeds contribute to temporal dispersion of germination (Li et al., 2005), thus avoiding the risk of simultaneous germination of the entire seed bank (Souza and Marcos-Filho, 2001). In wheat (*Triticum aestivum* L.), grain color gene (*R*) in red-grained line did not appear to increase the dormancy by accumulating germination inhibitors in bran, while sensitivity of embryo to ABA was higher in grains collected from red-grained and red-grained near-isogenic lines compared to white-grained and white-grained mutant lines (Himi et al., 2002). Similarly, it was found that under dry conditions, there was no significant difference between red-grained varieties Kitakei1354 (red dormant) and white-grained varieties AUS1408 (white dormant) in regard to seed dormancy, while a high correlation coefficient was detected between dormancy and embryonic sensitivity to ABA (Torada and Amano, 2002). However, under humid conditions, a red seed coat might be essential for maintaining high level of seed dormancy in wheat (Torada and Amano, 2002). These reports indicated that grain color gene should have a significant effect on seed dormancy by changing embryo sensitivity to ABA in wheat.

Besides the role of seed coat in a physical barrier, the impermeable seed coat may also play as a critical role in reducing the influx of numerous minerals and essential inorganic ions during seed germination and seed dormancy (Poljakoff-Mayber et al., 1994; Souza and Marcos-Filho, 2001). In a halophytic plant *Suaeda physophora*, it was observed that seed coat did not only restrict water uptake, but also reduced the influx of Na^+^ and Cl^−^ into embryo to prevent ionic toxicity (Song et al., 2007). Waxes are also important in making seedcoat impermeable to gasses and water, thus halophytes used waxes as impermeable substance that can prevent seed germination under stress conditions. In the black seeds of *S. salsa*, significant accumulation of waxes in seed coat was noted as compared with seed coats of brown seed, suggesting the protective role of waxes in protecting embryo from ionic toxicity (Song et al., 2017). The control of water absorption is also related to the hydrophobic nature of the testa (Souza and Marcos-Filho, 2001). Moreover waxy substances in black seeds inhibit water uptake and concomitantly, maintain seed viability for longer time than in brown seeds under high saline environment (Song et al., 2017).

Suberin is one of major lipid plyesters in seed coat that plays a very primitive role in seed germination. Beisson et al. (2007) reported that seed coats of *Arabidopsis* suberin mutants *gpat5* (Glycerol-3-phosphate acyltransferase 5) showed an abrupt increase in suberin accumulation in response to tetrazolium salt as compared with wild-type seed coats, which showed poor seed germination under salt stress. This provided the explicit role of *GPAT5* gene in suberin accumulation in seed coat and in seed germination under salt stress. In conclusion, seed coat is very important trait in understanding the seed germination pattern of dimorphic seeds under salt stress and halophytic species accumulate and deposit numerous inorganic and hydrophobic substances outside seed coat and/or in pallaside cells either to delay seed germination or to prevent seed germination under salt stress. Nonetheless, it has never been studied, how plants give signals to reproductive part to produce dimorphic seeds with thick seed coat. Therefore, future research should be considered this aspect and it will help to understand the role of seed coats in the adaptation of seed-heteromorphic halophytes to variable saline environments.

## Seed Dormancy in Heteromorphic Seeds

Seed dormancy is an important defense strategy in the heteromorphic seeds in halophytes. Seed dormancy governs germination pattern and germination timing under different environments, which also plays an important role in seed plant evolution and adaptation to environmental changes (Linkies et al., 2010). In halophytes, production of dormant seeds is also an important mechanism for survival under adverse environments and they produce dormant small seeds, which remain in soil un-germinated until salt concentration in the immediate vicinity of seed does not come under permissible salt concentration for optimum seed germination (Mandák and Pyšek, 2001; Li et al., 2005; Wang H.L. et al., 2012). One of the reasons could be associated with salt stress induced some signaling cascades which may trigger dormant seed production as an adaptive strategy in halophytes. Moreover under salt stress, high concentration of toxic ions (e.g., Na^+^) or production of reactive oxygen species may act as signaling molecules which may signal halophytic plants to produce heteromorphic seeds. A recent study highlighted this aspect and showed that with an increase in salt concentration from 1 to 500 mM NaCl stress level, *S. salsa* produced more brown seeds as compared with black seeds (Wang et al., 2015, 2018). Some external factors such as light and temperature and endogenous hormones level influence seed dormancy and germination in halophytes.

### Endogenous Phytohormones

Especially, ABA and GA (gibberellic acid), play important roles in seed dormancy. Generally, it is regarded that ABA and GA act antagonistically to each other; means ABA inhibits seed germination while GA improves seed germination (Kucera et al., 2005). Significant research has been conducted so far regarding exogenous application of hormones and seed germination-dormancy in plants under different environments (Li et al., 2005; Finch-Savage and Leubner-Metzger, 2006; Davies, 2010). However, little work has been done so far, showing the relation between endogenous hormones and dormancy/germination in heteromorphic seeds. In a study, it was observed that ZR, and ABA were more in brown seeds than black seeds in *Suaeda acuminata*; while IAA contents were higher in black seeds than brown seeds, which clearly suggested *S. acuminata* species accumulated ZR and ABA in tolerant seeds to delay seed germination under stress conditions for the survival of next generation (Wang H.L. et al., 2012). However this was not in case of *S. salsa*, in this halophytic species IAA, ZR, and ABA were more in brown seeds as compared with black seeds, and there was no significant difference in the content of GA between black and brown seeds (Wang et al., 2015). These results suggested that endogenous role of these hormones are not plant species specific, moreover different halophytic plant species may adopt different signaling pathways to interact with these hormones. It was also speculated that low germination in black seeds might be attributed to their higher ABA sensitivity rather than the difference in ABA content between black and brown seeds in *S. salsa* (Li et al., 2016). This indicates that interaction among endogenous hormones, especially ABA and GAs may play an important role in the dormancy status of heteromorphic seeds in these halophytes (Wang H.L. et al., 2012). Besides ABA and GA, other endogenous hormones also play roles in seed germination/dormancy. For example, ethylene may promote seed germination through the antagonism of ABA signaling (Xu et al., 2017). Likewise, BR (brassinosteroids) may also interact with other hormones and can influence seed dormancy. According to Steber and McCourt (2001) BR can increase seed germination by reducing ABA sensitivity. Therefore, the dormancy and germination trait was regulated by the interaction of these endogenous hormones in heteromorphic seeds.

### Light

It is an important factor which affects seed germination. Seed size also interferes with light. For example, small seeds are light sensitive which indicates that light acts as a depth-sensing mechanism, which usually avoids limited germination of seeds buried deep inside soil (Schutz et al., 2002). In *S. salsa*, black seeds germinated better in light as compared with brown seeds (Li et al., 2005). Rainfall in late summer may bring black seeds on the soil surface, and the germination of black seeds may start in *S. corniculata* (Cao et al., 2012). For *S. salsa*, brown seeds may germinate in spring when soil salinity is high, while black seeds may stay in soil and then germinate in later summer when rainfall can bring them on the soil surface (Li et al., 2005).

### Temperature

It is another environmental factor which interacts with salinity and affects seed germination. For example, in *Atriplex rosea*, black seeds are more sensitive to the change of temperature (Khan et al., 2004). Seed germination in black seeds decreased at lower temperature regimes regardless of salinity concentration, but brown seeds are more tolerant to temperature and salinity at cooler conditions (5/15°C). This was suggested that brown seeds may germinate early in the growing season to preempt the habitat for *A. rosea* (Khan et al., 2004). In *Salsola ferganica*, relatively lower daily temperature range, i.e., 5/15, 10/20, or 15/25°C could enhance germination of heteromorphic seeds (Ma et al., 2018). In *Atriplex centralasiatica*, the optimal temperature regime for black seed germination was 15°C while for brown seeds, it was 25°C. Moreover low salinity level did not influence the seed germination of black seeds under different temperature regimes (25/35°C), which showed that black seeds can germinate in the rainy summer season (Li et al., 2008). These results suggest that the response of dimorphic seeds to combined temperature and salinity could be an important strategy for dimorphic halophytes to survive in changeable saline environments.

## Growth and Reproduction Traits in the Descendants from Heteromorphic Seeds

**FIGURE 3:**
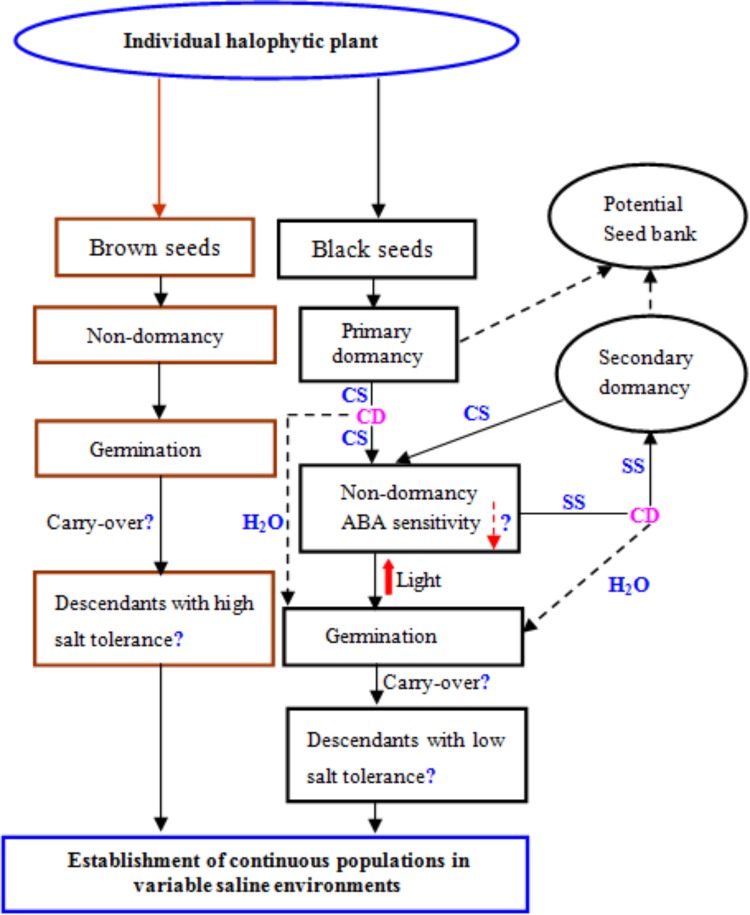
Conceptual model of the production of heteromorphic seeds for adaptation to saline environments in many halophytes, using dimorphic seeds as an example, which was modified from Wang et al. (2008). CS, cold tratification; CD, conditional dormancy; SS, salt stress; H_2_O, dilution by water. Up arrow indicates the increase and down arrow indicates the decrease.

*Chenopodium album* (a halophytic species) did not show any difference in germination and growth of plants developed from different seed dimorphs (Yao et al., 2010). In *Suaeda aralocaspica*, the descendants from heteromorphic seeds presented no significant difference in the osmolytes accumulation, activities of antioxidant enzymes, phosphoenolpyruvate carboxylase and the expression patterns of corresponding genes in performance with or without salinity (Cao et al., 2015). Moreover, shoot dry weight and the number of side branches along the main stem of plants from brown seeds were much higher than those of plants from black seeds in *S. salsa* under salinity (Liu et al., 2013). *Atriplex centralasiatica* produced two kinds of seed dimorphs such as yellow colored (which are tolerant to salt stress) and brown colored (which are salt sensitive) (Xu et al., 2011). Xu et al. (2011) showed that seedlings from yellow seeds exhibited better growth as compared with seedlings from brown seeds under salt stress and salt stress tolerance in seedlings from yellow colored seeds was associated with higher expressions of numerous genes relating to ion homeostasis, redox regulation, and hormones production. Moreover they also noted that seedlings developed from yellow seeds accumulated more nitric oxide (NO) as compared with seedlings from brown seeds and this was another reason how yellow seeds showed tolerance to salt stress as compared with brown seeds. Therefore, research is required to test the growth and reproduction traits in the descendants from heteromorphic seeds, using more seed-heteromorphic halophytes and providing more evidences whether there is carry-over of seed heteromorphism to plants. The conceptual model of the production of heteromorphic seeds for adaptation to saline environments in halophytes was shown in **Figure 3**.

## Conclusion

A series of papers mainly focus on the characteristics of dormancy and germination in heteromorphic seeds of halophytes. Only a few studies reported the different characteristics of heteromorphic seed development under different saline conditions, and the molecular evidence is scarce. Many halophytic plant species produce dimorphic seeds to ensure safe propagation of their subsequent generation. To do so, these species adopt different adaptive strategies and modify seed characteristics. Seed dormancy is one of major strategies in halophytes while producing dimorphic seeds, which makes them to stay longer under stress conditions. Maternal effects or transgenerational plasticity may also provide ecological diversity in the regenerative strategy for seed-heteromorphic halophytes. Role of seed coat and endogenous hormones is also very important relating to seed germination and seed dormancy. As future research perspective, we suggested to consider (1) electrophysiology of seed germination and cross talk between hormones and seed germination, and molecular evidences would be quite useful in understanding heteromorphism in halophytes, (2) it is interesting and unclear how an individual halophytic plant produces different types of seeds, and how environmental factors such as salinity affect their development, and (3) how heteromorphic seeds differently unload and release certain macroelements and micronutrients in the same maternal environment in halophytic species.

## Author Contributions

RL, LW, and JS wrote and revised the paper. MT revised the paper and polished the English.

## Conflict of Interest Statement

The authors declare that the research was conducted in the absence of any commercial or financial relationships that could be construed as a potential conflict of interest.
